# Characterization of *E93* in neometabolous thrips *Frankliniella occidentalis* and *Haplothrips brevitubus*

**DOI:** 10.1371/journal.pone.0254963

**Published:** 2021-07-22

**Authors:** Youhei Suzuki, Takahiro Shiotsuki, Akiya Jouraku, Ken Miura, Chieka Minakuchi

**Affiliations:** 1 Graduate School of Bio-Agricultural Sciences, Nagoya University, Nagoya, Japan; 2 Institute of Agrobiological Sciences, National Agriculture and Food Research Organization, Tsukuba, Japan; 3 Faculty of Life and Environmental Science, Shimane University, Matsue, Japan; Biocenter, Universität Würzburg, GERMANY

## Abstract

Insect metamorphosis into an adult occurs after the juvenile hormone (JH) titer decreases at the end of the juvenile stage. This generally coincides with decreased transcript levels of JH-response transcription factors *Krüppel homolog 1* (*Kr-h1*) and *broad* (*br*), and increased transcript levels of the adult specifier *E93*. Thrips (Thysanoptera) develop through inactive and non-feeding stages referred to as “propupa” and “pupa”, and this type of distinctive metamorphosis is called neometaboly. To understand the mechanisms of hormonal regulation in thrips metamorphosis, we previously analyzed the transcript levels of *Kr-h1* and *br* in two thrips species, *Frankliniella occidentalis* (Thripidae) and *Haplothrips brevitubus* (Phlaeothripidae). In both species, the transcript levels of *Kr-h1* and *br* decreased in the “propupal” and “pupal” stages, and their transcription was upregulated by exogenous JH mimic treatment. Here we analyzed the developmental profiles of *E93* in these two thrips species. Quantitative RT-PCR revealed that *E93* expression started to increase at the end of the larval stage in *F*. *occidentalis* and in the “propupal” stage of *H*. *brevitubus*, as *Kr-h1* and *br* mRNA levels decreased. Treatment with an exogenous JH mimic at the onset of metamorphosis prevented pupal-adult transition and caused repression of *E93*. These results indicated that E93 is involved in adult differentiation after JH titer decreases at the end of the larval stage of thrips. By comparing the expression profiles of *Kr-h1*, *br*, and *E93* among insect species, we propose that the “propupal” and “pupal” stages of thrips have some similarities with the holometabolous prepupal and pupal stages, respectively.

## Introduction

The manner of insect metamorphosis is classified into three major classes, i.e. ametaboly, hemimetaboly, and holometaboly. Among these classes, hemimetabolous insects develop directly from the nymph to the adult; the external morphology of hemimetabolous nymphs is similar to that of adults except for the absence of wings, and successive growth of wing pad is observed in the nymphal stage. Formation of the adult wings and genitalia is completed at the end of the nymphal stage. By contrast, holometabolous larvae develop to the adult through several larval instars and the pupal stage; degeneration of larval tissues and formation of adult structure proceed rapidly during the pupal stage. In addition to these categories, there is a unique type of metamorphosis called Neometaboly. Neometaboly, observed in thrips (Thysanoptera) and a part of hemipteran species such as whiteflies and male mealybugs, is defined by a life cycle in which feeding larvae develop to the adults via non-feeding stages with external wing primordia [1–3]. As is observed in other hemimetabolous species, the external morphology of the neometabolous nymphs resembles that of adults, but there are no external wing pads. Rapid proliferation of wing primordial cells starts at the end of the nymphal stage, then the wings become visible externally in the subsequent non-feeding stage. In the case of thrips, the inactive stage is called “propupa” and “pupa”, and they complete adult development through molting; the number of “propupal” and “pupal” instars varies among thrips species. The “propupa” and “pupa” of thrips look somewhat similar to pupae in holometabolous species, but unlike holometabolous pupae, “propupa” and “pupa” of thrips are mobile.

Insect molting and metamorphosis are generally regulated by molting hormone (ecdysone) and juvenile hormone (JH) [4–6]. In the post-embryonic development of holometabolous insects, JH is synthesized and secreted continuously through the penultimate larval instar, and pulses of ecdysone induce larva–larva molts in the presence of abundant endogenous JH. Once JH biosynthesis ceases in the final larval instar, ecdysone triggers larva–pupa and pupa–adult metamorphosis. A similar role of these hormones has been observed in hemimetabolous insects: ecdysone induces nymph–adult metamorphosis after JH titer decreases [7].

Transcription factors that mediate hormonal signaling in insect metamorphosis have been identified and characterized mainly in model insect species such as the fruit fly *Drosophila melanogaster*, the red flour beetle *Tribolium castaneum*, the silkworm *Bombyx mori*, and the cockroach *Blattella germanica*. *Krüppel homolog 1* (*Kr-h1*) was identified as an early response gene in JH signaling [8–10] downstream of JH receptor Methoprene-tolerant (Met)/Taiman (Tai) complex [11,12]. *Kr-h1* was reported as a repressor of metamorphosis activated by JH in a Met-dependent manner in hemimetabolous *Pyrrhocoris apterus* [13]. Subsequently, downstream target genes of Kr-h1 have been identified, including *broad* (*br*) [14] and *Ecdysone-induced protein 93F* (*E93*) [15]. *br* is a transcription factor that directs holometabolous pupal development [16–19] and promotes growth of wing pads in hemimetabolous species [19–21]. In general, the expression of *br* is induced by 20-hydroxyecdysone (20E), and this induction could also be affected by JH [19]. *br* expression in pupae could be induced by exogenous JH via *Met* and *Kr-h1* (Konopova and Jindra 2008; Minakuchi et al., 2009) [10,16]. In the silkworm *B*. *mori*, it was revealed that JH represses the 20E-mediated induction of *br* through the binding of Kr-h1 to its binding site (Kr-h1 binding site, KBS) in the *br* promoter [14]. Meanwhile, *E93*, an ecdysone-responsive, helix-turn-helix transcription factor with a Pipsqueak DNA-binding motif [22,23], was identified as a key factor that promotes adult development [24]. The role of *E93* in insect metamorphosis was first reported in *D*. *melanogaster*: *E93* is involved in salivary gland cell death in the prepupal stage in response to the ecdysteroid peak [22]. Functional analyses revealed that *E93* is essential for adult development in hemimetabolous cockroach *B*. *germanica*, holometabolous *D*. *melanogaster* and *T*. *castaneum* [24], as well as programmed cell death of nymphal tissues [25]. In *B*. *mori*, analysis of the promoter region of *E93* revealed that JH suppresses ecdysone-inducible *E93* transcription via Kr-h1 [15]. The genetic interaction between *Kr-h1* and *E93* has also been clarified in the hemimetabolous *B*. *germanica* and in the holometabolous *T*. *castaneum* [7,26]: *Kr-h1* suppresses the upregulation of *E93* in the juvenile stage, whereas *E93* represses *Kr-h1* expression in adult metamorphosis. Recently, expression analyses of *E93* revealed that the differential expression of *E93* is involved in sexual dimorphic development in the strepsipteran *Xenos vesparum* [27] as well as in the mealybug *Planococcus kraunhiae* [28]. The regulatory interaction among *Met*, *Kr-h1*, and *E93* is considered as the center of JH signaling, and called as ‘Met–Kr-h1–E93 (MEKRE93) pathway’ [7,29], or ‘Metamorphic Gene Network’ [26,30], which is thought to exist both in hemimetabolous and holometabolous species.

Thrips, categorized as a member of neometabolous species, develop through inactive non-feeding stages referred to as “propupa” and “pupa”. To understand the mechanisms of hormonal regulation in thrips metamorphosis, we previously examined the developmental expression profiles of *Kr-h1* and *br* in the western flower thrips *Frankliniella occidentalis* (Thripidae), which has one “propupal” instar and one “pupal” instar, and in *Haplothrips brevitubus* (Phlaeothripidae), which has one “propupal” instar and two “pupal” instars, i.e. pupa I and pupa II [31]. We revealed that the expression profiles of *Kr-h1* and *br* in the post-embryonic development are similar to those reported in holometabolous insect species: the transcript levels of *Kr-h1* and *br* decreased in the “propupal” and “pupal” stages, and that their transcription was upregulated by the presence of exogenous JH [31].

To understand the hormonal regulation of metamorphosis in thrips in more detail, we here analyzed the developmental expression profiles of *E93* in *F*. *occidentalis* and *H*. *brevitubus* by quantitative RT-PCR, and compared them with those of *Kr-h1* and *br*. We also analyzed the effects of treatment with an exogenous JH mimic (JHM) at the onset of metamorphosis on *E93* expression.

## Materials and methods

### Thrips rearing

*Frankliniella occidentalis* were provided by Professor T. Murai in Utsunomiya University. Larvae and adults were fed with germinated broad bean seeds (Kokusai Pet Food, Kobe, Japan) in plastic containers in accordance with a previously reported method [32], at 23±1°C with a long-day photoperiod (16L-8D). *Haplothrips brevitubus* were provided by Ishihara Sangyo Company (Osaka, Japan). They were raised with eggs of the Mediterranean flour moth *Ephestia kuehniella* (commercially available as Ento-food, from Arysta LifeScience, Tokyo, Japan) at 25±1°C with a long-day photoperiod (16L-8D) as reported previously [31].

### cDNA cloning

Total RNA was isolated from the whole body of *F*. *occidentalis* “propupae” and “pupae” using TRIzol reagent (Thermo Fisher Scientific, MA) with nuclease-free glycogen (Thermo Fisher Scientific) as a carrier, and first strand cDNA was synthesized using PrimeScript II reverse transcriptase with random hexamers (Takara Bio, Shiga, Japan). Primers for *F*. *occidentalis E93* (*FoE93*) were designed from our unpublished transcriptome database (RNA-seq). A part of *FoE93* cDNA was amplified based on an RT-PCR-approach using Tks Gflex DNA Polymerase (Takara Bio). Primer sequences are listed in S1 Table.

To amplify part of *H*. *brevitubus E93* (*HbE93*) cDNA, total RNA was extracted from the whole body of *H*. *brevitubus* using TRIzol reagent and nuclease-free glycogen as a carrier, and reverse-transcribed using a PrimeScript 1st strand cDNA Synthesis Kit with oligo-dT primers, as described previously [31]. Degenerate primers were designed based on the conserved amino acid sequences in the Pipsqueak DNA-binding motif [23] of other insects’ E93. PCR with HbE93-F1 and HbE93-R1 primers was performed at an annealing temperature of 50°C, followed by nested PCR with HbE93-F2 and HbE93-R2 primers at an annealing temperature of 40°C. Primer sequences are listed in S1 Table.

These PCR products were purified, cloned into a pGEM-T Easy vector (Promega) and sequenced. DNA sequence data have been deposited in the DDBJ/EMBL-Bank/GenBank International Nucleotide Sequence Database with the following accession numbers: LC415027 (HbE93) and LC415028 (FoE93).

### Quantitative RT-PCR analysis

**Fig 1:**
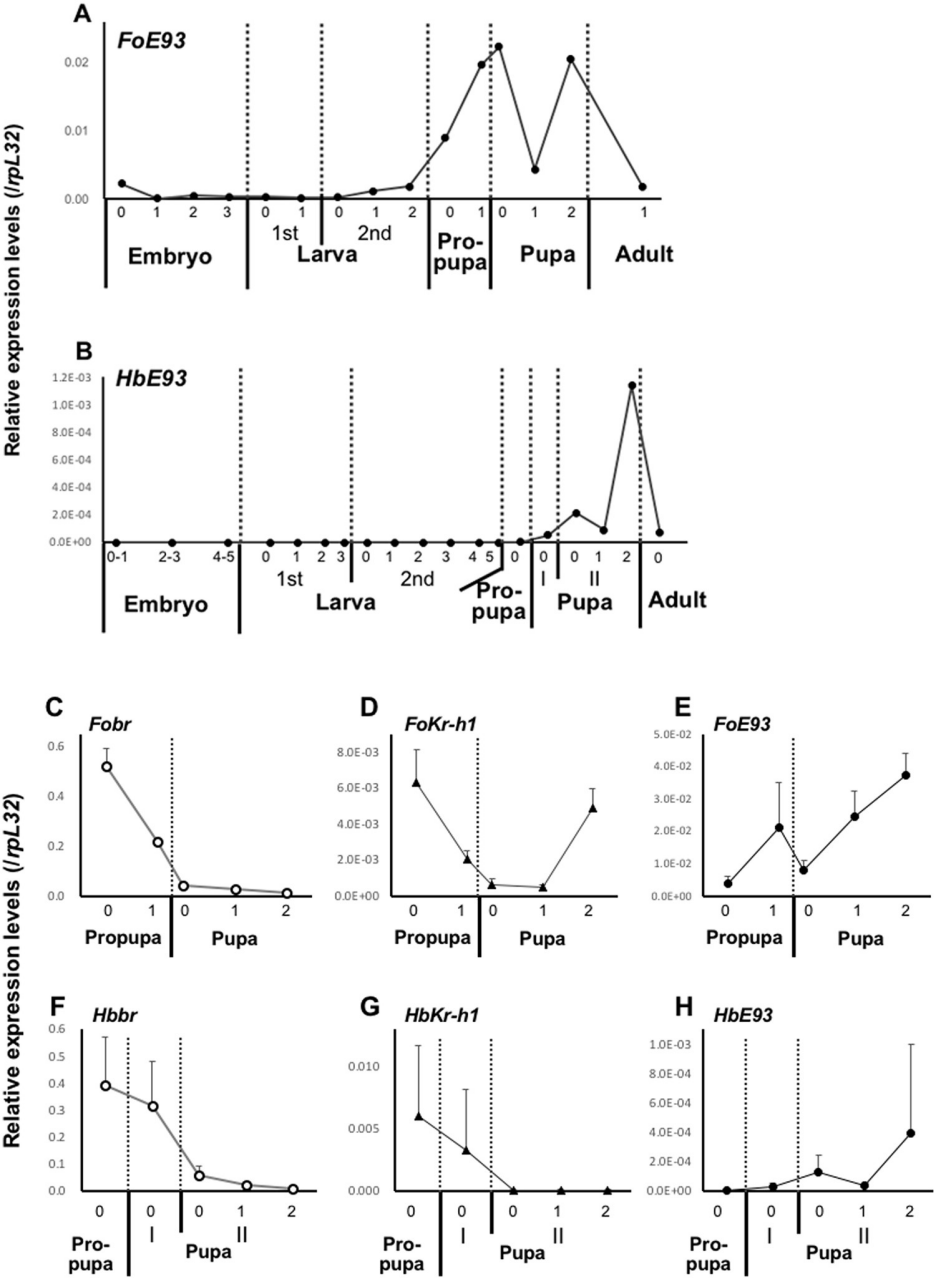
Developmental expression profiles of *F*. *occidentalis* (*Fo*) and *H*. *brevitubus* (*Hb*) *E93*. Transcript levels of *E93* were determined by quantitative RT-PCR, and the values were normalized to those of *rpL32*. Total RNA was isolated from a pool of individuals for each time point. Numbers on the abscissa indicate the ages in days. (A) Developmental expression profiles of *FoE93*. (B) Developmental expression profiles of *HbE93*. In (A) and (B), the same set of cDNAs as in our previous study [31] was used (*n* = 1). (C–E) Expression profiles of *Fobr* (C), *FoKr-h1* (D), and *FoE93* (E) in “propupal” and “pupal” stages. (F–H) Expression profiles of *Hbbr* (F), *HbKr-h1* (G), and *HbE93* (H) in “propupal” and “pupal” stages. In (C–H), means and standard deviations are shown (*n* = 3).

To analyze the developmental expression profiles and the transcript level after JHM treatment using quantitative RT-PCR, cDNA synthesis was performed as reported previously [31]. Briefly, a bunch of staged animals were pooled for RNA isolation at each time point. To analyze the transcript level in JHM-treated *F*. *occidentalis* or *H*. *brevitubus* (see below), 8–14 individuals were pooled for RNA isolation. Total RNA was isolated from the whole body using TRIzol reagent with RNase-free glycogen (Thermo Fisher Scientific) as a carrier, treated with DNase I (Takara Bio), and reverse-transcribed using a PrimeScript 1st Strand cDNA Synthesis Kit with an oligo-dT primer. Alternatively, some RNA samples were reverse transcribed using a PrimeScript RT reagent Kit with gDNA Eraser (Takara Bio; results shown in Fig 1C–1H).

The transcript levels were quantified using a real-time thermal cycler (Thermal Cycler Dice TP800, Takara Bio). Quantitative RT-PCR was carried out in a 14-μl reaction volume containing SYBR Premix Ex Taq (Takara Bio), 0.2 μM of each primer (see S1 Table), and 1 μl of template cDNA or standard plasmids. PCR conditions were 95°C for 30 s, followed by 40–45 cycles at 95°C for 5 s and 60°C for 30 s. After the thermal cycling, the absence of unwanted byproducts was confirmed by melting curve analysis. Serial dilutions of a plasmid containing a part of the ORF of each gene were used as standards. Analysis was performed two or three times for each sample, and the average was calculated. The transcript levels of *br*, *Kr-h1*, and *E93* were normalized to that of *rpL32* in the same samples. Primer sequences are listed in S1 Table.

### Hormonal treatments

Juvenile hormone mimic (JHM) treatment was performed as reported previously [31]. Briefly, a JHM, pyriproxyfen, was dissolved in methanol at a concentration of 3.2 μg/ml (10 mM). To examine the effect of JHM on the expression of *E93*, newly molted *F*. *occidentalis* “propupae” within 8 h after ecdysis were anesthetized with ether for 1.5–2 min, were dipped into 10 mM pyriproxyfen or methanol as a control for 10 sec, and total RNA was extracted 48 h later when they were on Day 0 of the pupal stage. Four to 7 animals were pooled for RNA isolation in each replicate, and treatments were performed in 4 biological replicates.

Newly molted *H*. *brevitubus* “propupae” within 5 h after ecdysis were anesthetized on ice, dipped in 10 mM pyriproxyfen or methanol as a control, and total RNA was extracted 72 h later when they were on Day 1 of the Pupa II stage, as described previously [31]. Eight to 11 animals were pooled for RNA isolation in each replicate, and treatments were performed in 2 biological replicates.

In both species, a few animals that died immediately after the treatment, due to the toxicity of the solvent (less than 5% of total animals tested), were excluded from the experiments. Quantitative RT-PCR was conducted as described above.

### Statistical analyses

Multiple comparison was performed by one-way analysis of variance (ANOVA) and Tukey-Kramer method by using the software GraphPad Prism version 8.

## Results and discussion

### cDNA sequences of *E93* of *F*. *occidentalis* and *H*. *brevitubus*

Tblastn searches of the transcriptome database of *F*. *occidentalis* identified a contig homologous to *B*. *germanica* E93 (accession number, CCM97102), and RT-PCR primers were designed to amplify part of the *E93* cDNA. A 788-bp fragment was amplified by RT-PCR with FoE93-fwd1 and FoE93-rev1 primers. The nucleotide sequence of this fragment was identical to that obtained from the transcriptome. Blastx searches of this partial sequence identified a protein sequence of *F*. *occidentalis* E93 (XP_026276121.1, annotated as “mushroom body large-type Kenyon cell-specific protein 1”), which was not yet registered when we started cDNA cloning. Multiple alignment of E93 sequences revealed that *F*. *occidentalis* E93 (XP_026276121.1) was homologous to E93 of other insects, being the Pipsqueak DNA binding motif specially well conserved (S1 Fig).

A 142-bp partial fragment for *E93* was amplified by nested RT-PCR with HbE93-F2 and HbE93-R2 primers from the cDNA pool of *H*. *brevitubus*. Blastx searches showed that this was homologous to counterparts in other insects. Multiple alignment is shown in S2 Fig.

### Developmental expression profile of *E93* mRNA

The transcript levels of *E93* were measured by quantitative RT-PCR. In *F*. *occidentalis*, *FoE93* mRNA remained low until the end of the larval stage, increased rapidly in the “propupal” stage, and decreased in the adult stage (Fig 1A). In *H*. *brevitubus*, *HbE93* mRNA remained low from the embryonic stage until the “propupal” stage, and increased in the “pupa I” and “pupa II” stages (Fig 1B).

We showed previously that the expression profiles of *Kr-h1* and *br* in post-embryonic development in thrips have some similarities to those reported in holometabolous insect species [31]. In thrips, the transcript level of *Kr-h1* is maintained in the larval stage, whereas *br* is highly expressed from the beginning of the last larval instar until the “propupal” stage (diagram of the expression profile is shown in Fig 3B). Although JH titer has not been measured in thrips due to their small size, we estimate based on the expression profile of *Kr-h1* that JH titer decreases in the “propupal” stage. In this study, we compared the expression profiles of *br*, *Kr-h1*, and *E93* in the “propupal” and “pupal” stages. In both species, the transcript levels of *Kr-h1* and *br* decreased at the end of the “propupal” stage as *E93* increased (Fig 1C–1H). This observation was also consistent with the findings in holometabolous insects: the peak of *Kr-h1* and *br* decreased at pupation, whereas *E93* increased at the beginning of the pupal stage [26].

### Effects of JHM treatment on the expression of *br*, *Kr-h1*, and *E93* in adult development

**Fig 2:**
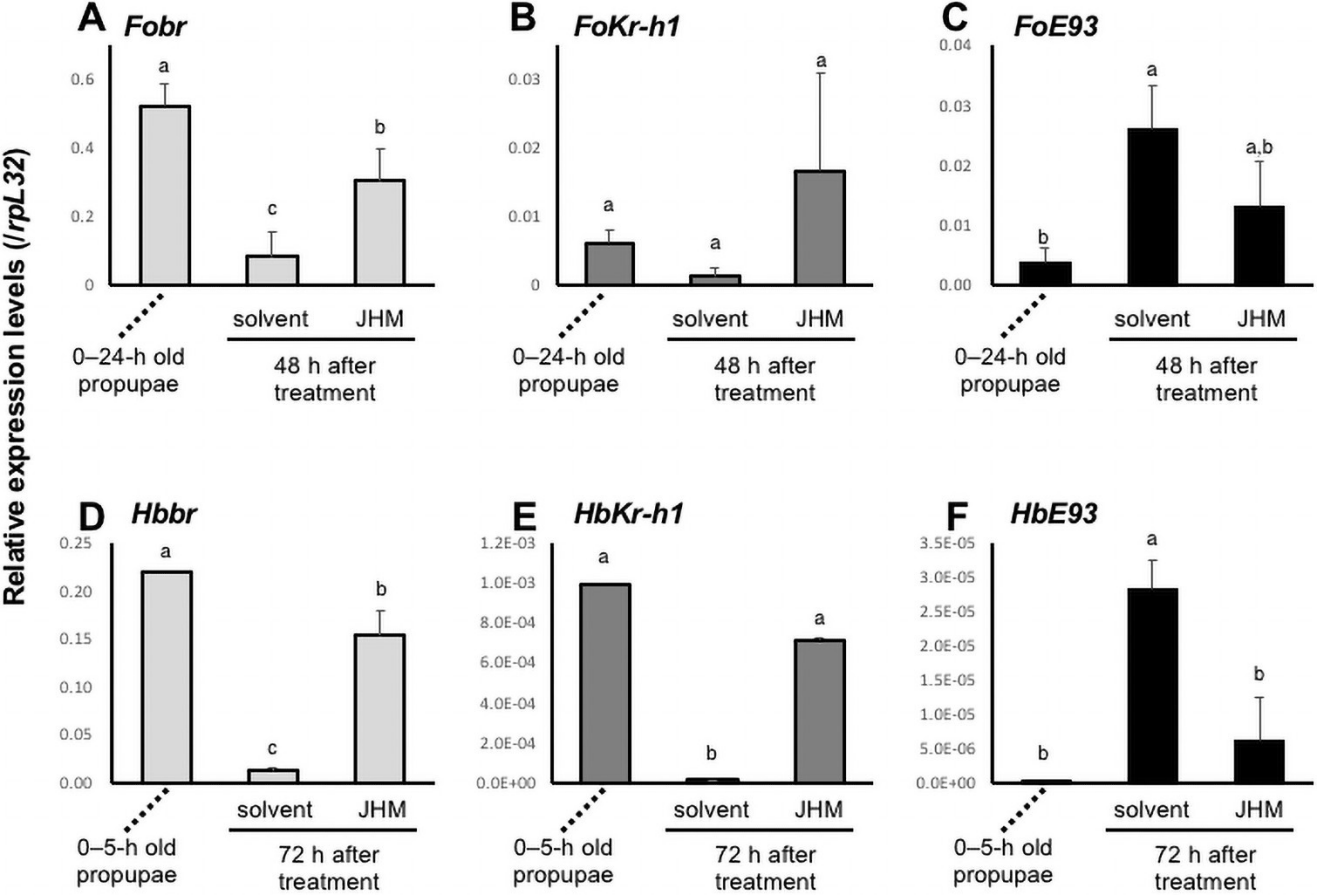
Transcript levels of *br*, *Kr-h1*, and *E93* after JHM (10 mM pyriproxyfen) treatment. Means with the same letter are not significantly different (Tukey–Kramer test, P < 0.05). (A–C) Newly molted “propupae” of *F*. *occidentalis* were treated with JHM or solvent only, and the *Fobr* (A), *FoKr-h1* (B), and *FoE93* (C) transcripts were quantified 48 h later. Pools of 4–7 animals were used for RNA isolation in each replicate, and means and standard deviations from 4 biological replicates are shown. The values were normalized to those of *ForpL32*. The transcript levels in 0–24 h-old “propupae” were also shown. (D–F) Newly molted “propupae” of *H*. *brevitubus* were treated with JHM or solvent only, and the *Hbbr* (D), *HbKr-h1* (E), and *HbE93* (F) transcripts were quantified 72 h later. Pools of 8–11 animals were used for RNA isolation in each replicate, and means and standard errors from 2 biological replicates are shown. The values were normalized to those of *HbrpL32*. The transcript levels in 0–5 h-old “propupae” are also shown.

We reported previously that treating “propupae” of *F*. *occidentalis* or *H*. *brevitubus* with exogenous JHM prevented pupa–adult metamorphosis; treatment of *F*. *occidentalis* “propupae” at 0–18 h after ecdysis with 10 mM pyriproxyfen resulted in lethality in “propupa” (6%), “pupa” (91%) and newly-ecdysed adults (3%), while treatment of “propupae” with 10 mM pyriproxyfen caused lethality in “propupa” (17%), “pupa I” (17%), “pupa II” (62%) and newly-ecdysed adults (4%) [31]. Moreover, JHM treatment caused prolonged expression of *Kr-h1* and *br* [31]. Here, we examined the transcript level of *br*, *Kr-h1*, and *E93* after JHM treatment of newly-molted “propupae”. In *F*. *occidentalis*, *Fobr* was upregulated by JHM treatment compared with solvent-treated individuals (Fig 2A), whereas the upregulation of *FoKr-h1* was not statistically significant (Fig 2B). The level of the *FoE93* transcript in control animals increased 48 h after solvent treatment compared with that in 0–24-h old “propurae”, whereas that in JHM-treated “pupae” was slightly lower (Fig 2C). Similarly, we examined the transcript level of *br*, *Kr-h1*, and *E93* in *H*. *brevitubus*. *Hbbr* and *HbKr-h1* were upregulated by JHM treatment compared with solvent-treated individuals (Fig 2D and 2E). The transcript level of *HbE93* in JHM-treated “pupae” was suppressed compared with that in solvent-treated “pupae” (Fig 2F). Thus, JHM treatment to the “propupa” resulted in upregulation of *br* and *Kr-h1*, and suppression of *E93* in *H*. *brevitubus*. Although similar effects were observed in *F*. *occidentalis*, it seemed to be less effective compared with *H*. *brevitubus*. It is possible that the response to exogenous JHM is different between these two species. Alternatively, this might be due to difference in time points for qRT-PCR: transcript levels were examined at 48 h after treatment in *F*. *occidentalis*, while they were examined at 72 h after treatment in *H*. *brevitubus*.

### Possible regulatory mechanism of *E93* transcription in thrips

**Fig 3:**
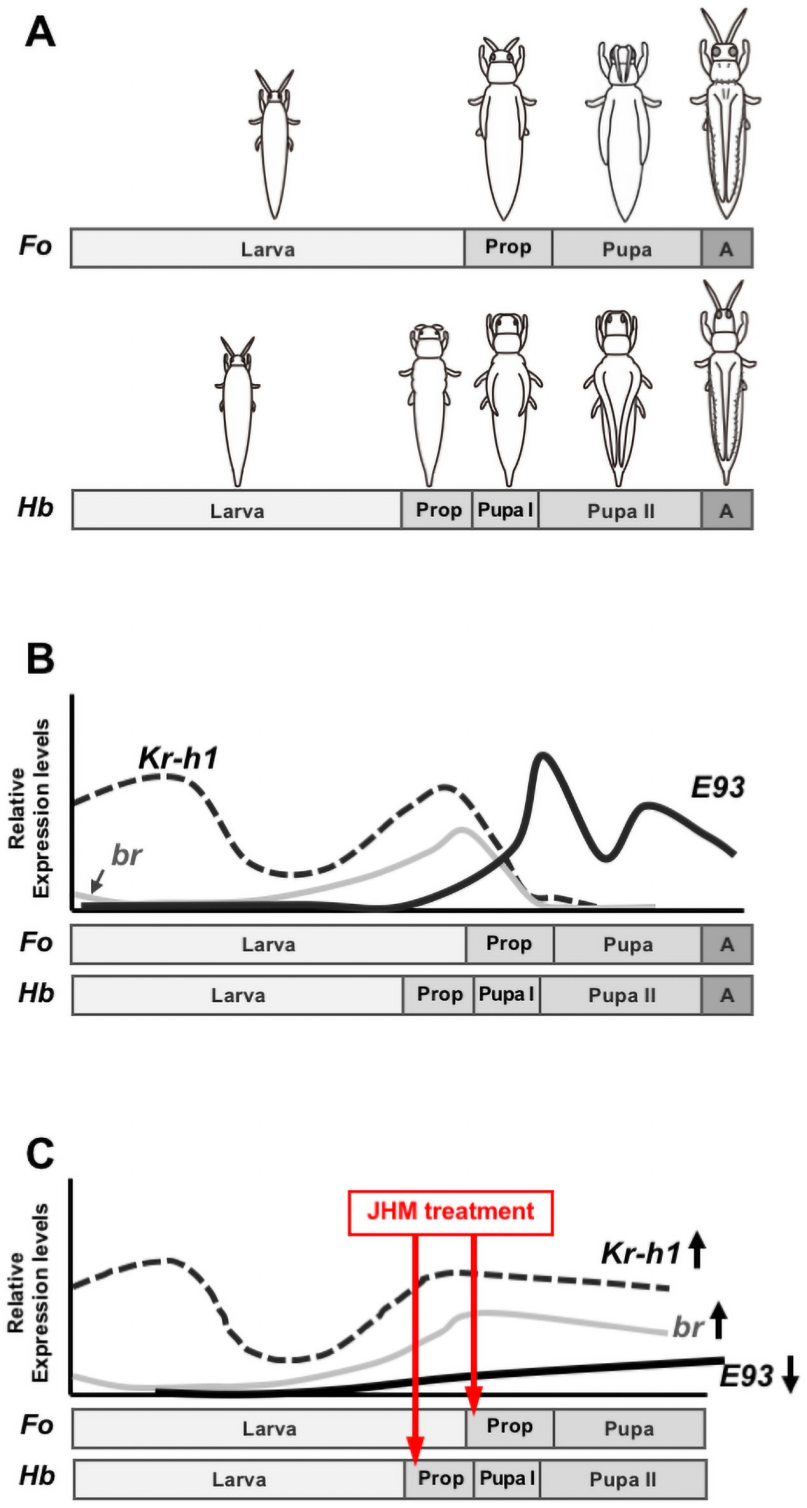
Diagram of the expression profiles of the selected transcription factors in thrips. (A) Postembryonic development of the two thrips species, *Frankliniella occidentalis* (abbreviated as *Fo*) and *Haplothrips brevitubus* (*Hb*). (B and C) Diagram of the expression profiles of *br*, *Kr-h1*, and *E93*. The expression patterns of *br* and *Kr-h1* were illustrated based on our previous report (Minakuchi et al., 2011) [31]. (B) In normal thrips, *E93* levels increase as *Kr-h1* and *br* levels decrease. (C) After exogenous JHM treatment at the onset of metamorphosis, the expression of *Kr-h1* and *br* is prolonged, whereas *E93* induction is suppressed.

The regulatory mechanism of *E93* transcription was first elucidated in *B*. *germanica*, *T*. *castaneum*, *D*. *melanogaster*, and *B*. *mori* [7,15,24,26,30]. *E93* has also been characterized in other species including paraneopteran brown planthopper *Nilaparvata lugens* [33] and common bed bug *Cimex lectularius* [34]. In hemimetabolous *B*. *germanica*, endogenous JH, through Kr-h1, prevented 20E-induced upregulation of *E93* in the nymphal stage. Once JH titer decreased at the onset of nymph–adult metamorphosis, increasing levels of *E93* induced adult differentiation, and downregulation of *Kr-h1* and *br* expression levels. A similar interaction was observed in holometabolous *T*. *castaneum* and *D*. *melanogaster* [24,26]. In *T*. *castaneum*, a transient peak in *Kr-h1* in the prepupal stage blocked precocious upregulation of *E93* in the larva–pupa transition [26]. After pupation, with low JH titer, 20E-inducible *E93* directed adult differentiation and suppressed *Kr-h1* and *br* [24]. A similar interaction among *Kr-h1*, *br*, and *E93* has been confirmed in *D*. *melanogaster* [24,26]. In *B*. *mori*, JH suppressed 20E-inducible *E93* expression, and this suppression was mediated by Kr-h1 through its binding to a Kr-h1 binding site located in the *E93* promoter region [15]. In this study, we were not able to clarify the regulatory interaction among *Kr-h1*, *br*, and *E93* in thrips, because RNAi-mediated knockdown was not applicable. However, judging from the expression profiles of *Kr-h1*, *br*, and *E93* (Figs 1 and 2) [31], we propose that their regulatory interaction may be conserved in thrips as well. A small peak of *Kr-h1* exists in the “propupal” stage of thrips, which coincides with the peak of *br* (Fig 3B). We propose that JH in the “propupal” stage of thrips prevents precocious induction of *E93* through *Kr-h1* and *br*, thus preventing precocious adult differentiation. Normally, adult differentiation is induced by *E93* with low levels of *Kr-h1* and *br* in the absence of JH. If JHM was applied at the beginning of the “propupal” stage, the expression of *Kr-h1* and *br* was prolonged and the expression of *E93* was downregulated, which suppressed adult development (Fig 3C). Thus, although the regulatory interactions among *Kr-h1*, *br*, and *E93* remain unclear, it is very likely that the MEKRE93 pathway [7] is conserved in thrips, too.

Thrips develop through an inactive non-feeding stage referred to as “propupa” and “pupa”, after the larval stage. The significance of these inactive, pupa-like stages has been discussed. A part of larval tissues degenerates at the larva-pupa transition, whereas the adult structure is formed by newly-proliferating cells. Degeneration of the larval muscles was observed in the “propupal” and “pupal” stages of *Frankliniella fusca* and *Haplothrips verbasci* [35]. As stated above, the overall developmental expression profiles of *Kr-h1* and *br* in thrips (Fig 3B) are similar to that of holometabolous species rather than that of hemimetabolous species [7,30]: the expression of *br* is maintained in the nymphal stage in Hemimetabola, whereas *br* is specifically expressed at larva-pupa transition of Holometabola and thrips. In this study, we showed that the transcript level of *E93* increased as those of *Kr-h1* and *br* decreased in the “propupal” stage of thrips (Fig 3B). This observation was also consistent with the findings in holometabolous insects: the peak of *Kr-h1* and *br* decreased at pupation, whereas *E93* increased at the beginning of the pupal stage. We propose that the “propupal” stage of thrips is similar to the holometabolous prepupal stage just before pupation, and that the “propupal” stage of thrips is essential for the formation of “pupa”. Also, we propose that the “pupal” stage of the thrips has similarities with the pupal stage in holometabolous insects. In Hemimetabola, it has been suggested that the final nymphal instar is ontogenetically homologous to holometabolous pupal stage; this was first proposed by Hinton [36] and has been supported by others [7,13,37]. From the expression profiles of *Kr-h1*, *br*, and *E93*, we propose that the “propupal” and “pupal” stages in thrips is similar to the final nymphal instar in hemimetabolous species.

In conclusion, we revealed the developmental expression profiles of *E93* in two thrips species. The results indicated that E93 induces adult differentiation after JH titer decreases at the end of the larval stage of thrips. We propose that the role of *E93* in adult differentiation and hormonal regulation of its transcription are conserved among a variety of insect species including thrips. In addition, we propose that the “propupal” stage of thrips has similarities with the holometabolous prepupal stage, whereas the “pupal” stage of the thrips is similar to the pupal stage in holometabolous insects. Our findings will contribute significantly to solving the mystery of how metamorphosis in thrips evolved.

## Supporting information

S1 Fig Alignment of protein sequences of E93. The protein sequences of E93 were aligned using Clustal Omega (https://www.ebi.ac.uk/Tools/msa/clustalo/). DmE93, *Drosophila melanogaster* E93 (accession number, NP_652002.2); FoE93, *Frankliniella occidentalis* E93 (XP_026276121.1); BmE93, *Bombyx mori* E93 (AIL29268.1); TcE93, *Tribolium castaneum* E93 (XP_015839257.1); BgE93, *Blattella germanica* E93 (CCM97102.1). Putative Pipsqueak DNA-binding domain is boxed. Three symbols below aligned sequences indicate fully (*), strongly (:) and weakly (.) conserved residues, respectively.
(TIFF)

S2 Fig Alignment of protein sequences of E93 Pipsqueak DNA-binding domains. The protein sequences of E93 were aligned using Clustal Omega (https://www.ebi.ac.uk/Tools/msa/clustalo/). Fo, *Frankliniella occidentalis*; Hb, *Haplothrips brevitubus*; Tc, *Tribolium castaneum* (XP_015839257.1); Bm, *Bombyx mori* (AIL29268.1); Bg, *Blattella germanica* (CCM97102.1); Dm, *Drosophila melanogaster* (NP_652002.2). Dashes in HbE93 sequence represent the part where nucleotide sequence has not been analyzed since RACE PCR has not been performed for *H*. *brevitubus E93*. Three symbols below aligned sequences indicate fully (*), strongly (:) and weakly (.) conserved residues, respectively.
(TIFF)

S1 Table Primer sequences for RT-PCR and qRT-PCR. (XLSX)
