# Supplementary figures and images for: Characterization of E93 in neometabolous thrips Frankliniella occidentalis and Haplothrips brevitubus
Source: PLoS One. 2021 Jul 22;16(7):e0254963. doi: 10.1371/journal.pone.0254963 (PMC8297894; doi:10.1371/journal.pone.0254963)

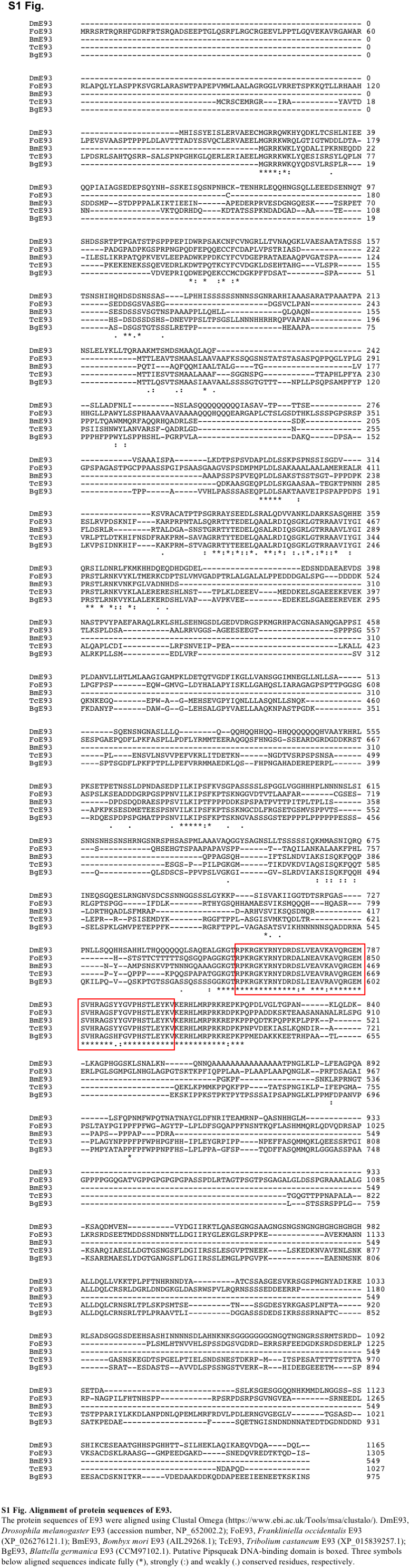

Supplement: S1 Fig — The protein sequences of E93 were aligned using Clustal Omega (https://www.ebi.ac.uk/Tools/msa/clustalo/). DmE93, Drosophila melanogaster E93 (accession number, NP_652002.2); FoE93, Frankliniella occidentalis E93 (XP_026276121.1); BmE93, Bombyx mori E93 (AIL29268.1); TcE93, Tribolium castaneum E93 (XP_015839257.1); BgE93, Blattella germanica E93 (CCM97102.1). Putative Pipsqueak DNA-binding domain is boxed. Three symbols below aligned sequences indicate fully (*), strongly (:) and weakly (.) conserved residues, respectively. (TIFF) [file pone.0254963.s001.tiff]

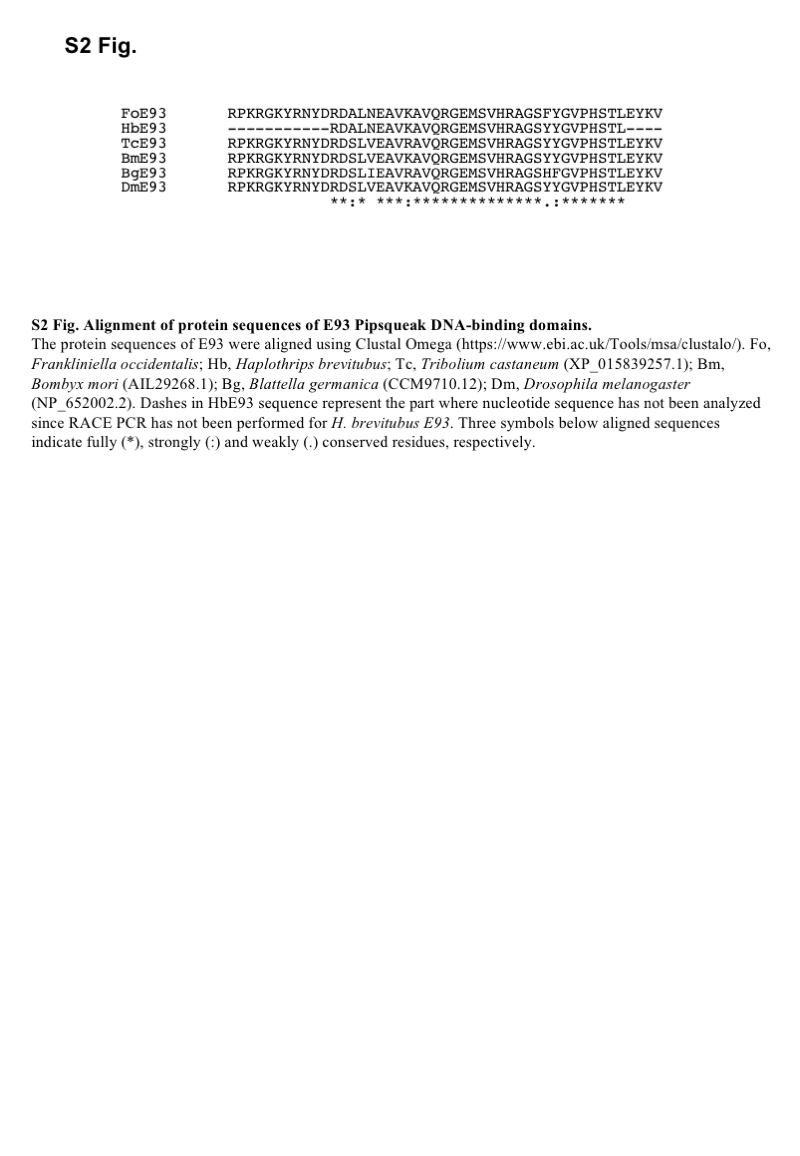

Supplement: S2 Fig — The protein sequences of E93 were aligned using Clustal Omega (https://www.ebi.ac.uk/Tools/msa/clustalo/). Fo, Frankliniella occidentalis; Hb, Haplothrips brevitubus; Tc, Tribolium castaneum (XP_015839257.1); Bm, Bombyx mori (AIL29268.1); Bg, Blattella germanica (CCM97102.1); Dm, Drosophila melanogaster (NP_652002.2). Dashes in HbE93 sequence represent the part where nucleotide sequence has not been analyzed since RACE PCR has not been performed for H. brevitubus E93. Three symbols below aligned sequences indicate fully (*), strongly (:) and weakly (.) conserved residues, respectively. (TIFF) [file pone.0254963.s002.tiff]
